# Profiling severe asthma: Any relevance for age? An analysis from Severe Asthma Network Italy (SANI) cohort^☆^

**DOI:** 10.1016/j.waojou.2024.100941

**Published:** 2024-07-29

**Authors:** Marco Caminati, Alessandro Marcon, Rachele Vaia, Gianenrico Senna, Matteo Maule, Pierpaolo Marchetti, Jessica Miotti, Giuseppe Argentino, Francesco Blasi, Giorgio W. Canonica, Enrico M. Heffler, Pierluigi Paggiaro, Andrea Vianello, Gabriella Guarnieri, The SANI study group, Luisa Brussino, Cecilia Calabrese, Gianna Camiciottoli, Giovanna E. Carpagnano, Stefano Centanni, Angelo G. Corsico, Maria T. Costantino, Claudia Crimi, Alice D'adda, Simona D'alo, Maria D'amato, Stefano Del Giacco, Fabiano Di Marco, Nicola C. Facciolongo, Manuela Latorre, Eustachio Nettis, Eleonora Nucera, Giovanni Passalacqua, Girolamo Pelaia, Laura Pini, Luisa Ricciardi, Luca Richeldi, Erminia Ridolo, Pierachille Santus, Nicola Scichilone, Giulia Scioscia, Giuseppe Spadaro, Antonio Spanevello, Paolo Tarsia

**Affiliations:** j Immunologia Clinica e Allergologia, Ospedale Mauriziano, Torino, Italy; k Department of Translational Medical Sciences, University of Campania “Luigi Vanvitelli”, Naples, Italy; l UNIT ASMA GRAVE - Ambulatorio Asma Grave Pneumologia e Fisiopatologia Toraco-Polmonare, Azienda Ospedaliera Universitaria Careggi (FI), Florence, Italy; m Department of Translational Biomedicine and Neuroscience“DiBraiN”, University of Bari Aldo Moro, Bari, Italy; n Respiratory Unit, ASST Santi Paolo e Carlo, San Paolo Hospital, Department of Health Sciences, University of Milan, Milan, Italy; o Division of Respiratory Diseases, IRCCS Policlinico San Matteo, Foundation and Department of Internal Medicine and Therapeutics, University of Pavia, Italy; p Allergy and Clinical Immunology Unit, Department of Medicine, “Carlo Poma” Hospital Mantova, Italy; q Respiratory Medicine Unit, Policlinico “G. Rodolico-San Marco” University Hospital, Catania, Italy; r Broncopneumologia, Fondazione IRCCS Ca’ Granda Ospedale Maggiore Policlinico, Milan, Italy; s UOC Allergologia PO Civitanova Marche AREA VASTA 3, Civitanova Marche, Italy; t Department of Respiratory Medicine, University “Federico II” of Naples, Italy; u Unit of Allergy and Clinical Immunology, Department of Medical Sciences and Public Health, University of Cagliari, Cagliari, Italy; v Pulmonary Medicine Unit, ASST Papa Giovanni XXIII Hospital, Bergamo, Italy; w UOC di Pneumologia, Arcispedale S. Maria Nuova Azienda USL di Reggio Emilia, Italy; x Pulmonary Unit, Nuovo Ospedale Apuano, Massa, Italy; y Department of Emergency and Organ Transplantation, School and Chair of Allergology and Clinical Immunology, University of Bari, Bari, Italy; z Fondazione Policlinico Universitario Agostino Gemelli IRCCS - Università Cattolica del Sacro Cuore Roma, Italy; aa UOSD Allergologia e Immunologia Clinica, Dipartimento Scienze Mediche e Chirurgiche, Fondazione Policlinico Universitario A. Gemelli IRCCS Roma, Italy; ab Clinica di Malattie Respiratorie e Allergologia, Dip. Medicina Interna, Univ. degli Studi di Genova, IRCCS-AOU San Martino, Genoa, Italy; ac U.O. Malattie dell’Apparato Respiratorio, A.O.U. Mater Domini, Catanzaro, Italy; ad Department of Clinical and Experimental Sciences, University of Brescia - ASST Spedali Civili di Brescia, Italy; ae Allergologia e Immunologia Clinica, AOU Policlinico G. Martino - Università di Messina, Messina Italy; af Fondazione Policlinico Universitario A. Gemelli, IRCCS Catholic University of Rome, Italy; ag Department of Medicine and Surgery, University of Parma, Parma, Italy; ah Department of Clinical and Biomedical Sciences, University of Milan, Respiratory Diseases, Sacco University Hospital, ASST Fatebenefratelli-Sacco, Milan, Italy; ai U.O.C. Pneumologia, Azienda Ospedaliera Universitaria Policlinico P. Giaccone di Palermo, Italy; aj Malattie Apparato Respiratorio, Dipartimenti delle funzioni Mediche e Sanitarie, Azienda Ospedaliero Universitaria - Ospedali Riuniti, Foggia, Italy; ak Center for Basic and Clinical Immunology Research (CISI), University of Naples Federico II, Naples, Italy; al Pneumologia Riabilitativa, ICS Maugeri IRCCS Tradate, Tradate (VA), Italy; am Pneumology Unit, Grande Ospedale Metropolitano Niguarda, Milan, Italy; an Section of Respiratory Diseases, Policlinico Hospital of Bari, Italy; a Department of Medicine, University of Verona, Verona, Italy; b Department of Diagnostics and Public Health, Unit of Epidemiology and Medical Statistics, University of Verona, Verona, Italy; c Asthma Center and Allergy Unit, Verona University Hospital, Verona, Italy; d Internal Medicine Department, Respiratory Unit and Cystic Fibrosis Center, Fondazione IRCCS Cà Granda Ospedale Maggiore Policlinico di Milano, Milan, Italy; e Department of Pathophysiology and Transplantation, Università degli Studi di Milano, Milan, Italy; f Department of Biomedical Sciences, Humanitas University, Pieve Emanuele, Milan, Italy; g Personalized Medicine, Asthma and Allergy, Humanitas Clinical and Research Center, IRCCS, Rozzano, Milano, Italy; h Department of Surgery, Medicine, Molecular Biology and Critical Care, University of Pisa, Pisa, Italy; i Department of Cardiac, Thoracic and Vascular Sciences, University of Padova, Padova, Italy

**Keywords:** Severe asthma, Aging, Comorbidities, Lung function, Asthma control

## Abstract

**Background:**

Aging implies changes in terms of lung function, immune system, and respiratory and extra-respiratory comorbidities. Few studies have specifically addressed the relevance of age on severe asthma burden and control. We aimed to evaluate whether age acts as an independent determinant of asthma severity, in terms of clinical, functional, and inflammatory profile, and to explore potential cofactors that contribute to a more difficult disease control in different age groups.

**Methods:**

Patients from Severe Asthma Network Italy (SANI) registry were retrospectively divided in subgroups according to their age. Cutoffs for age were established according to quartiles in order to obtain a comparable number of patients for each group, and then rounded for the sake of simplicity.

**Results:**

Overall, 1805 severe asthma patients were analyzed. Lung function represented the most important age-related variable. On the opposite the level of asthma control was not differently distributed among age ranges. In young people the presence of atopy-related comorbidities (allergic rhinitis, atopic dermatitis) predominated, whilst systemic-metabolic and degenerative comorbidities such as diabetes, cardiovascular diseases, anxious-depressive syndrome, and osteoporosis prevailed in elderly. Bronchiectasis and sleep disturbances were significantly associated with age.

**Conclusions:**

Despite that it cannot be considered a treatable trait, our study suggests that age should be evaluated within a personalized approach to severe asthma patients, in order to provide a better clinical profiling and a more tailored treatment strategy.

## Background

Assessing the inflammatory pheno-endotype of severe asthma patients is a critical issue in their management.^1^ However, a proper approach to the disease should not be limited to the identification of major inflammation drivers, as well as the definition of T2-high or T2-low pattern, as other factors might affect the clinical course.^2^ Recently, the treatable traits approach has been introduced as a step forward towards personalized medicine. Despite it cannot be considered a treatable trait, age might have an impact on asthma at different levels, in terms of comorbidities as well as progressive impairment of lung function and immune response.^3^, ^4^, ^5^ In addition, as the proportion of the world's population over 45 years is projected to increase rapidly, investigation of the preventable age-related determinants of severe asthma, and treatment of the reversible age-related risk factors that contribute to severe asthma, should be part of a tailored clinical and therapeutic approach. This study aimed to evaluate whether age acts as an independent determinant of asthma severity, in terms of clinical, functional, and inflammatory profile. The analysis also explored potential cofactors associated to patients' age and potentially contributing to a more difficult disease control in different age sub-groups.

## Methods

Data were collected from Severe Asthma Network Italy (SANI) registry. SANI is a web-based observatory that collects demographic, clinical, and functional data, as well as inflammatory biomarkers of patients with severe asthma, defined according to European Respiratory Society (ERS)/American Thoracic Society (ATS) classification and aged >12 years, recruited by accredited centers homogeneously spread out on the national territory. Considering the real-life perspective provided by SANI registry, no exclusion criteria (including the possibility to have received a diagnosis of asthma-COPD overlap) were present in the protocol. Information collected in the SANI registry include demographic, clinical (ie, allergic sensitizations, comorbidities, information on asthma exacerbations, asthma control, asthma-related quality of life), functional (lung function parameters), inflammatory (ie, blood eosinophils, serum IgE, exhaled nitric oxide) and asthma-related treatment data.^6^

Patients were divided in subgroups according to their age. However, as exploring the relevance of age as an independent determinant of asthma severity and burden was the primary outcome of the study, the population was not clustered according to pre-determined age-ranges, but age distribution within the sample was observed. In particular, cutoffs for age were established according to quartiles in order to obtain a comparable number of patients for each group for statistical reasons, and then rounded for the sake of simplicity. Clinical, functional, and inflammation related variables were considered (Table 1, Table 2, Table 3). More in detail, BMI, smoking habits, age at asthma onset, disease duration, asthma severity according to GINA classification, disease control (patient's reported outcomes and exacerbations requiring oral corticosteroids [OCS]), and both respiratory and extra respiratory comorbidities were analyzed as part of the patient's clinical profile. In terms of lung function, pre- and post-bronchodilator parameters were considered. In order to assess inflammatory features peripheral eosinophils and neutrophils count, total serum IgE concentration, and FeNO measurement were included. According to the secondary outcome of the study, which was investing potential cofactors associated to patients' age and potentially contributing to a more difficult disease control in different age sub-groups, patients' characteristics were then compared between age groups using mean ± SD and median (1st quartile [Q1]–3rd quartile [Q3]) for symmetrical and asymmetrical quantitative variables, respectively, and proportions (%) for categorical variables; we tested differences between groups using the Kruskal-Wallis test for quantitative variables, Chi squared and Fisher's exact tests for categorical variables as appropriate.

**Table 1:** Patients’ characteristics, by age group.

Characteristic	Data available (n)	Overall (n = 1805)	Age 14-44 y (n = 335)	Age 45-54 y (n = 447)	Age 55-64 y (n = 574)	Age 65-86 y (n = 449)	p-value^b^
Female, n (%)	1805	1113 (61.7)	208 (62.1)	280 (62.6)	356 (62.0)	269 (59.9)	0.84
Ethnicity, n (%)							
Caucasian	1758	1706 (97.0)	303 (93.5)	421 (96.6)	546 (97.7)	436 (99.3)	<0.001
Other		52 (3.0)	21 (6.5)	15 (3.4)	13 (2.3)	3 (0.7)	
Height, mean ± SD (cm)	1747	165.3 (±9.7)	167.7 (±8.9)	167.1 (±9.7)	164.6 (±9.5)	162.8 (±9.8)	<0.001
BMI, mean ± SD (kg/m^2^)	1745	26.3 (±5.0)	25.0 (±5.2)	25.7 (±4.6)	26.7 (±5.0)	27.2 (±4.9)	<0.001
Smoking habits, n (%)							
Non-smoker	1772	1242 (70.1)	229 (69.6)	326 (73.8)	393 (69.8)	294 (67.1)	<0.001
Ex-smoker		461 (26.0)	70 (21.3)	100 (22.6)	155 (27.5)	136 (31.0)	
Current smoker		69 (3.9)	30 (9.1)	16 (3.6)	15 (2.7)	8 (1.8)	
Pack Years, median (Q1-Q3)^a^	500	10 (5–20)	5.0 (2.0–10.0)	10.0 (4.0–20.0)	10.2 (5.3–20.0)	10.0 (6.0–25.0)	<0.001
Age at asthma onset, mean ± SD (y)	1618	33.4 (±16.8)	20.1 (±11.0)	30.0 (±14.0)	36.4 (±15.1)	43.2 (±17.5)	<0.001
Age at asthma diagnosis, mean ± SD (y)	1638	35.8 (±16.8)	22.2 (±10.9)	31.7 (±13.9)	39.0 (±14.8)	46.0 (±17.6)	<0.001
Disease duration, median (Q1-Q3) (y)	1606	19.0 (10.0–32.0)	12.0 (6.0–22.0)	18.0 (8.0–32.0)	20.0 (11.0–34.0)	25.0 (14.0–40.0)	<0.001
GINA classification, n (%)							
Step 4	1587	220 (13.9)	42 (14.0)	54 (13.5)	70 (14.0)	54 (13.9)	0.99
Step 5		1367 (86.1)	258 (86.0)	345 (86.5)	429 (86.0)	335 (86.1)	
Disease control, n (%)							
Uncontrolled asthma	1589	1306 (82.2)	249 (83.0)	319 (80.1)	414 (83.1)	324 (82.4)	0.66
Controlled asthma		283 (17.8)	51 (17.0)	79 (19.8)	84 (16.9)	69 (17.6)	
Frequent exacerbations requiring OCS, n (%)	1151	946 (82.2)	184 (82.5)	236 (84.9)	281 (79.4)	245 (82.8)	0.34

Abbreviations: BMI: Body Mass Index; GINA: Global Initiative for Asthma; OCS: OCS: Oral Corticosteroids; SD: Standard Deviation.

a Calculated among current and ex-smokers.

b Kruskal-Wallis test for quantitative variables, chi-square or Fisher's exact test for categorical variables

**Table 2:** Patients’ comorbidities, by age group.

Comorbidity	Data available (n)	Overall (n = 1842)	Age 14-44 y (n = 335)	Age 45-54 y (n = 447)	Age 55-64 y (n = 574)	Age 65-86 y (n = 486)	P-value^c^
Allergic rhinitis, n (%)	1746	769 (44.0)	174 (53.4)	199 (45.8)	232 (42.0)	164 (37.8)	<0.001
Perennial allergic rhinitis, n (%)^a^	769	575 (74.8)	135 (77.6)	152 (76.4)	162 (69.8)	126 (76.8)	0.22
Seasonal allergic rhinitis, n (%)^a^	769	224 (29.1)	52 (29.9)	57 (28.6)	74 (31.9)	41 (25.0)	0.52
Chronic rhinosinusitis without nasal polyps, n (%)	1702	496 (29.1)	82 (25.6)	146 (34.4)	151 (28.1)	117 (27.9)	0.044
Nasal polyposis last 12 months, n (%)	1761	777 (44.1)	124 (37.7)	201 (46.0)	262 (46.8)	190 (43.7)	0.051
Underwent polypectomy last 12 months, n (%)^b^	571	454 (79.5)	68 (79.1)	126 (79.8)	152 (80.0)	108 (78.8)	0.99
Sleep quality last 12 months, n (%)							
Nothing to report	1648	1209 (73.4)	242 (79.9)	304 (73.8)	363 (68.9)	300 (73.9)	0.006
Snoring		347 (21.1)	50 (16.5)	93 (22.6)	126 (23.9)	78 (19.2)	
OSAS		92 (5.6)	11 (3.6)	15 (3.6)	38 (7.2)	28 (6.9)	
Bronchiectasis, n (%)	1472	314 (21.3)	34 (12.4)	70 (20.1)	103 (21.8)	107 (28.5)	<0.001
Atopic dermatitis, n (%)	1758	119 (6.8)	39 (11.9)	31 (7.2)	22 (3.9)	27 (6.2)	<0.001
ASA/NSAID hypersensitivity, n (%)	1734	281 (16.2)	46 (14.3)	64 (14.9)	85 (15.5)	86 (19.8)	0.13
GERD diagnosis, n (%)							
No	1744	1075 (61.6)	215 (67.0)	278 (64.2)	330 (59.5)	252 (57.9)	0.048
Confirmed		482 (27.6)	68 (21.2)	111 (25.6)	166 (29.9)	137 (31.5)	
Suspected		187 (10.7)	38 (11.8)	44 (10.2)	59 (10.6)	46 (10.6)	
Obesity (BMI ≥30 kg/m^2^), n (%)	1745	352 (20.2)	47 (14.5)	73 (17.0)	126 (22.6)	106 (24.4)	0.001
Cardiovascular disease, n (%)	1609	437 (27.2)	20 (6.5)	70 (17.4)	160 (32.1)	187 (46.6)	<0.001
Anxious-depressive syndrome, n (%)	1598	155 (9.7)	14 (4.6)	29 (7.4)	61 (12.2)	51 (12.8)	<0.001
Type-2 diabetes, n (%)	1617	87 (5.4)	3 (1.0)	9 (2.2)	37 (7.3)	38 (9.5)	<0.001
Peptic ulcer, n (%)	1600	24 (1.5)	0 (0.0)	7 (1.7)	7 (1.4)	10 (2.5)	0.023
Osteoporosis, n (%)	1457	229 (15.7)	9 (3.0)	36 (10.0)	83 (18.7)	101 (28.6)	<0.001

Abbreviations: ASA: Acetylsalicylic Acid; NSAID: Non-Steroid Anti-Inflammatory Drugs; BMI: Body Mass Index; GERD: Gastroesophageal Reflux Disease; OSAS: Obstructive Sleep Apnoea Syndrome.

a Calculated among patients with allergic rhinitis.

b Calculated among patients with nasal polyposis in the last 12 months.

c Kruskal-Wallis test for quantitative variables, chi-square or Fisher's exact test for categorical variables

**Table 3:** Patients’ clinical characteristics, by age group.

	Data available (n)	Overall (n = 1842)	Age 14–44 y (n = 335)	Age 45–54 y (n = 447)	Age 55–64 y (n = 574)	Age 65–86 y (n = 486)	p-value^a^
Absolute eosinophil count, median (Q1-Q3)	1387	0.4 (0.1–0.7)	0.4 (0.1–0.7)	0.4 (0.1–0.7)	0.4 (0.1–0.7)	0.3 (0.1–0.6)	0.68
Percentage eosinophil count, median (Q1-Q3)	1254	4.4 (1.7–8.6)	4.0 (1.6–8.0)	4.8 (1.9–8.8)	4.6 (1.6–9.0)	4.3 (1.6–8.3)	0.49
Absolute neutrophil count, median (Q1-Q3)	1070	4.0 (3.1–5.3)	4.2 (3.2–5.6)	4.0 (3.2–5.3)	3.8 (3.0–5.0)	4.2 (3.1–5.6)	0.046
Percentage neutrophil count, median (Q1-Q3)	1033	55.3 (48.6–62.1)	56.8 (49.9–63.5)	54.5 (48.3–61.9)	54.2 (47.6–60.0)	55.5 (48.5–62.9)	0.057
Higher blood eosinophil count, median (Q1-Q3)	1013	0.6 (0.3–1.1)	0.6 (0.3–1.3)	0.7 (0.4–1.1)	0.6 (0.3–0.9)	0.6 (0.3–1.0)	0.19
Total serum IgE concentration, median (Q1-Q3)	1080	198 (77.4–483.9)	223.0 (87.4–580.5)	193.0 (71.0–416.0)	180.5 (71.5–440.5)	233.0 (93.0–511.0)	0.34
Chest CT last 2 years, n (%)							
No	1665	903 (54.2)	185 (59.7)	247 (59.5)	266 (50.0)	205 (50.2)	<0.001
Yes, normal		300 (18.0)	59 (19.0)	77 (18.5)	100 (18.8)	64 (15.7)	
Yes, altered		462 (27.8)	66 (21.3)	91 (21.9)	166 (31.2)	139 (34.1)	
FVC pre-BD (L), mean ± SD	1303	3.1 (±1.0)	3.7 (±0.9)	3.3 (±0.9)	3.0 (±0.9)	2.6 (±0.9)	<0.001
FVC % predicted pre-BD, mean ± SD	1288	91.4 (±20.0)	92.2 (±16.8)	91.8 (±19.4)	91.3 (±20.8)	90.4 (±21.5)	0.37
FVC post-BD (L), mean ± SD	499	3.2 (±1.0)	3.8 (±0.9)	3.4 (±0.9)	3.0 (±0.9)	2.7 (±0.8)	<0.001
FVC % predicted post-BD, mean ± SD	490	93.8 (±19.0)	94.6 (±16.6)	92.6 (±17.9)	94.2 (±20.0)	94.0 (±20.5)	0.88
FEV_1_ pre-BD (L), mean ± SD	1308	2.1 (±0.8)	2.6 (±0.8)	2.3 (±0.8)	2.0 (±0.7)	1.6 (±0.7)	<0.001
FEV_1_% predicted pre-BD, mean ± SD	1300	75.9 (±21.2)	78.9 (±19.0)	76.9 (±20.5)	76.4 (±22.3)	72.2 (±21.7)	<0.001
FEV_1_ post-BD (L), mean ± SD	673	2.2 (±0.8)	2.8 (±0.8)	2.4 (±0.7)	2.0 (±0.7)	1.8 (±0.6)	<0.001
FEV_1_% predicted post-BD, mean ± SD	654	81.0 (±20.7)	85.1 (±19.1)	81.0 (±19.2)	79.8 (±21.6)	79.3 (±22.0)	0.013
FEV_1_/FVC pre-BD, mean ± SD	1295	67.6 (±12.2)	71.9 (±12.2)	69.0 (±11.4)	67.5 (±11.9)	63.3 (±12.0)	<0.001
FEV_1_/FVC post-BD, mean ± SD	494	68.7 (±12.4)	73.6 (±11.4)	70.2 (±11.0)	68.4 (±12.4)	64.3 (±12.8)	<0.001
Tiffeneau index % predicted post-BD, mean ± SD	320	75.7 (±17.1)	80.2 (±15.8)	77.1 (±18.1)	78.1 (±16.1)	66.7 (±15.6)	<0.001
FeNO (ppb), median (Q1-Q3)	828	30.0 (16.0–55.0)	31.0 (15.0–62.0)	30.5 (16.0–61.5)	31.2 (17.0–56.0)	25.5 (14.0–50.0)	0.22
ACT, median (Q1-Q3)	1586	18.0 (13.0–22.0)	18.0 (14.0–22.0)	18.5 (13.0–22.0)	18.0 (13.0–22.0)	18.0 (14.0–22.0)	0.99
ACQ, median (Q1-Q3)	1183	2.3 (1.0–3.4)	2.2 (1.0–3.2)	2.3 (1.0–3.8)	2.3 (1.0–3.6)	2.3 (1.1–3.4)	0.58
AQLQ Score, median (Q1-Q3)	1339	4.5 (3.5–5.8)	4.6 (3.6–5.8)	4.4 (3.4–5.8)	4.5 (3.6–5.8)	4.5 (3.4–5.7)	0.49
Symptom score, median (Q1-Q3)	1336	4.5 (3.3–5.8)	4.6 (3.4–5.9)	4.3 (3.3–5.9)	4.4 (3.3–5.8)	4.5 (3.3–5.8)	0.73
Activity limitation score, median (Q1-Q3)	1339	4.5 (3.5–5.7)	4.8 (3.7–6.0)	4.5 (3.5–5.7)	4.6 (3.6–5.7)	4.5 (3.4–5.5)	0.11
Emotional function score, median (Q1-Q3)	1334	4.6 (3.4–6.2)	4.8 (3.6–6.0)	4.4 (3.4–6.0)	4.6 (3.4–6.2)	4.6 (3.4–6.2)	0.67
Environmental stimulus score, median (Q1-Q3)	1334	4.5 (3.3–5.8)	4.5 (3.5–5.5)	4.3 (3.3–5.8)	4.5 (3.3–5.5)	4.3 (3.3–6.0)	0.78
N. days of work lost last 12 months, n (%)							
0	1059	665 (62.8)	108 (53.7)	149 (55.2)	202 (59.9)	206 (82.1)	<0.001
1–7		134 (12.6)	34 (16.9)	44 (16.3)	41 (12.2)	15 (6.0)	
>7		260 (24.6)	59 (29.4)	77 (28.5)	94 (27.9)	30 (12.0)	
N. days subtracted from free time last 12 months, n (%)							
0	1009	543 (53.8)	94 (48.2)	136 (52.7)	164 (52.4)	149 (61.3)	0.10
1–7		113 (11.2)	29 (14.9)	31 (12.0)	34 (10.9)	19 (7.8)	
>7		353 (35.0)	72 (36.9)	91 (35.3)	115 (36.7)	75 (30.9)	
≥1 admission in the emergency room last 12 months, n (%)	1605	269 (16.8)	65 (22.3)	63 (15.7)	73 (14.2)	68 (17.0)	0.027
≥1 unscheduled visit last 12 months, n (%)	1382	408 (29.5)	77 (30.9)	101 (28.5)	128 (28.9)	102 (30.4)	0.90
N. exacerbations with steroid use last 12 months, n (%)							
0	1534	521 (34.0)	77 (27.9)	133 (33.9)	178 (37.0)	133 (34.6)	0.21
≤2		482 (31.4)	95 (34.4)	132 (33.7)	139 (28.9)	116 (30.1)	
>2		531 (34.6)	104 (37.7)	127 (32.4)	164 (34.1)	136 (35.3)	

Abbreviations: ACQ: Asthma Control Questionnaire; ACT: Asthma Control Test; AQLQ: Asthma Quality of Life Questionnaire; BD: Bronchodilator; CT: Computed Tomography; FeNO: Fractional Exhaled Nitric Oxide; FEV1: Forced Expiratory Volume in the first second; FVC: Forced Vital Capacity; IgE: Immunoglobulin E.

a Kruskal-Wallis test for quantitative variables, chi-square or Fisher's exact test for categorical variables

## Results

Overall, 1805 patients affected by severe asthma were analyzed. Table 1 shows patients’ characteristics by age group. Patients were aged 14–86 years old. Females represented 61.7% (n 1113) of the cohort. Most patients were Caucasian (97%). Regarding ethnicity, we observed a higher number of patients belonging to non-Caucasian ethnic groups in younger patients. Mean body mass index (BMI) was 26.3 (±5.0) and showed a gradual increase from young patients to the elderly.

Most patients in our population were not active smokers, but among smokers, active ones were significantly more frequent in younger age groups, whereas the proportion of ex-smokers was higher in the older groups.

Late onset asthma was more commonly referred by elderly patients, who also presented a longer disease duration and an older age at diagnosis when compared to youngest.

Interestingly, no significant differences in asthma severity and control were found between age groups. In particular, the proportion of patients diagnosed with GINA step 4 or 5 was comparable across different age groups. No significant differences between different age groups were found in asthma control in terms of exacerbation rate.

Table 2 reports patients’ comorbidities by age group. Allergic rhinitis was significantly more prevalent among younger patients, as well as atopic dermatitis. Our data showed a homogeneous distribution of perennial and seasonal allergic sensitization between groups, suggesting there is no significant difference in sensitization profile between age groups.

Interestingly, no significant different distribution of chronic rhinosinusitis with and without nasal polyps was found between age groups. On the opposite, the presence of sleep disturbances seemed to increase with age. In our population, snoring was significantly more frequent in subjects older than 45, and obstructive sleep apnea syndrome (OSAS) in older than 55. Similarly, the coexistence of gastroesophageal reflux disease (GERD) showed a linear increase with age, as well as bronchiectasis. ASA/NSAIDs hypersensitivity seems to equally affect younger and older patients. Cardiovascular diseases, anxious/depressive syndrome, type-2 diabetes and osteoporosis were more prevalent in older patients. The distribution trend of obesity seemed to parallel aging.

Functional, clinical and immunological data for age group were evaluated as described in Table 3. Laboratory data, including blood eosinophils, neutrophils, and total serum IgE, did not differ among age groups. Another marker of T2-high inflammation, exhaled nitric oxide (FeNO), showed a homogeneous distribution as well.

CT scan was more likely to be abnormal in older patients compared to younger ones, consistently with the distribution of bronchiectasis reported in Table 2.

When looking at lung function assessment, significantly lower values were observed in terms of FVC, FEV1 and Tiffenau Index in elderly.

Of note, patient-reported outcomes and exacerbations defined as emergency room (ER) access or not scheduled follow-up visits did not follow the same distribution. In our population, no significant difference was found in these parameters among age groups, suggesting that a worse lung function is not always correlated with a worse symptom control neither with a higher number of exacerbations.

## Discussion

Our study investigated the relevance of age as an independent determinant of asthma severity by analyzing severe asthma features in different age groups, and by evaluating the prevalence of asthma comorbidities in different ages. According to that purpose, and differently from other reports,^3^^,^^5^^,^^7^^,^^8^ the population was not clustered according to pre-determined age-ranges, but cutoffs were established according to age distribution in our sample.

When looking at patient's clinical profile, our data confirm in the largest Italian elderly asthma population sample some expected and already described features,^3^^,^^5^^,^^7^, ^8^, ^9^, ^10^ related to longer disease duration and the recurrence of some specific comorbidities aged patients. In young people the presence of atopy-related comorbidities (allergic rhinitis, atopic dermatitis) predominates, consistently with published data, confirming that early onset asthma is more often atopy-related.^7^

With aging, systemic-metabolic and degenerative comorbidities such as diabetes, cardiovascular diseases, anxious-depressive syndrome, and osteoporosis prevailed. Of note, most of the recurring comorbidities are part of the known OCS-related adverse events^11^ and might be hypothesized that it is the case of their origin in our population, due to the long disease duration, the limited availability of alternative treatment options in the past, and age-related steroid resistance development as part of immune-senescence.^8^^,^^10^ However, excluding OCS from the therapy of elderly asthmatic should be considered a major goal.

Of note, we found that bronchiectasis also significantly associated with age and were characterized by a higher incidence in adults >45 years. Such comorbidity has not been specifically focused by other reports;^3^^,^^5^^,^^7^, ^8^, ^9^, ^10^ however it is even more relevant in the elderly, not only as a known risk factor for uncontrolled asthma,^12^ but also because bronchiectasis further increasing susceptibility to infections, which is also connected with immune-senescence.^8^^,^^10^ For those reasons bronchiectasis should be regularly assessed through high resolution computed tomography (HRCT) and properly addressed as a treatable trait in all adult asthma patients and especially in the elderly. Besides macrolide antibiotics,^13^ some emerging data suggest some benefit form specific biologic treatment.^14^

Obesity prevalence was also significantly higher in elderly, as confirmed by body mass index (BMI), which tends to increase with aging and this component must undoubtedly be valued in clinical practice. Of note, obesity, and dis-metabolic conditions, when characterizing patients affected by diseases chronically requiring steroid treatment, should be considered in the light of corticosteroid-related comorbidities and properly addressed by optimizing asthma treatment itself.^15^ Other comorbidities associated with asthma unexpectedly showed a comparable distribution among the different age groups: chronic rhinosinusitis with or without nasal polyposis, non-steroid anti-inflammatory drugs (NSAIDs) hypersensitivity, and sleep disorders. Those conditions are known determinants of worse asthma control, and some authors suggested that specifically targeting such comorbidities substantially contributes to asthma control. Their homogeneous distribution within the various age groups is therefore extremely relevant and suggests including the investigation of those conditions in the checklist of severe asthma patients' assessment regardless their age. When moving to lung function parameters, they expectedly represent the most important age-related variable. In fact, the physiological decline in respiratory mechanics observed with ageing, combined with a potentially longer disease duration in elderly,^4^^,^^16^ account for that. Of note, when looking at patients' reported symptoms and exacerbation indicators, the proportion of subjects characterized by poor asthma control was not related to age, suggesting that the relevance of spirometry evaluation as part of the disease assessment and treatment response is crucial independently of patients’ age, but when interpreting the spirometry results, disease duration and physiological decline in lung function should be taken into consideration especially in the elderly. The assessment of the response to specific asthma treatments or the definition of asthma remission should also be tailored according to the same background when evaluating lung function improvement.^17^ The above-mentioned observations seem to confirm what is reported in the literature about the need for a detailed analysis of spirometry in old age, and about the possibility of identifying other functional indicators different from the traditional ones (FVC, FEV1, Tiffenau Index) that might have a stronger correlation with disease control.^18^ Finally, regarding the relationship between phenotyping and endotyping, and the use of inflammation biomarkers, our analysis only partially reflects what is reported in the literature. Juvenile asthma, as already mentioned, is more frequently related to atopic diseases,^19^ sharing with them a common T2 immune background. However, our data showed that there is no statistically significant difference in blood eosinophilic or neutrophilic counts between different age groups. In particular, there was neither higher blood eosinophils in younger age groups compared to the elderly, nor an increase in neutrophilic count in the elderly compared to the young. The same observation emerged from the analysis of FeNO and total serum IgE, which did not show higher values in the younger age groups. The discrepancy between our observation and other reports in the literature might rely on the relatively young age of asthma onset, with high intra-individual variability, within the oldest subgroup of our population sample. In fact it has been suggested that a long-standing asthma is more often characterize by a T2 background also in elderly.^10^

However, on one hand the presented data are consistent with the indication to always evaluate T2 related biomarkers in asthmatic patients, regardless of their clinical history and comorbidities. On the other hand, they seem to confirm that, although the understanding of the pathophysiological mechanisms of asthma has progressed considerably in recent years, the simplest and commonly used biomarkers are not uniquely related to the presence of T2-high or T2-low inflammation, or that there are other mechanisms that interfere with this relationship.^20^^,^^21^ However, since the aim of this study was to analyze the characteristics of patients in relation to age, this evaluation went beyond the objective of the investigation. In addition, the lack of data on sputum eosinophils and neutrophils, which seem to have a more relevant diagnostic correlation with inflammation endotype,^22^ could represent a limitation concerning the investigation of T2 biomarkers. On the other hand, sputum collection and processing are not routinely performed by all the referral centers for severe asthma, due to complex procedure and the need for dedicated personnel.

Generally speaking, asthma in the elderly used to be considered a more difficult to control disease, being associated with a higher rate of healthcare costs when compared to the same condition in younger individuals.^8^^,^^10^ Of note, according to our findings, controlled/uncontrolled asthma proportion, the mean patients reported outcomes scores, and the rate of asthma exacerbations requiring OCS are equally distributed across all the subgroups of our sample, and, unfortunately, it's not due to an overall increase of optimally controlled patients. Both our observations and the evidence from the literature suggest common and different age related-determinants, the last including longer disease duration, bronchiectasis, systemic comorbidities, inflammaging processes and immune-senescence for the elderly group.^8^^,^^10^ On the opposite side, we demonstrated a higher prevalence of active smoking in the 14–45 years subgroup. However, although age cannot be considered a treatable trait itself, still it associates with age-specific comorbidities and determinants of higher disease burden that deserve to be investigated, assessed and properly treated according to an age-tailored approach.

## Conclusions

Our study aimed to provide additional tools that allow the clinician a better profiling of the patient suffering from severe asthma. Much has been said in the literature about the need for proper phenotyping and endotyping of patients, and about the use of biomarkers that help clinicians in this process. Less amount of evidence has been provided about the clinical profiling of the patient in particular in relation to age.

What our study highlights is that from a clinical point of view the greatest difference between young and old asthma patients is represented by lung function aspects. This would suggest that a timely intervention could prevent the worsening of lung function; on the other hand, the evaluation of lung function as a major treatment outcome might be not appropriate in the elderly.

The preserved lung function in the youngest should be considered a strong rationale for an early tailored pharmacological intervention as well as education on potential determinants of asthma worsening, including smoking habits. In fact, we observed a higher prevalence of active smoking habit in the 14–45 years subgroup, which deserved to be specifically addressed.

The other aspect that differentiates the approach to elderly patients from the young ones is the evaluation of intra- and extra-respiratory comorbidities, which have a different profile by age group. However, it is useful to consider that some comorbidities related to a worse control of the disease must be investigated and treated regardless the patient age, such as NSAIDs hypersensitivity, chronic rhinosinusitis, and sleep disorders.

Finally, the search for T2 related biomarkers should always be performed, regardless of age, history of atopic comorbidities, and smoking history, given their relevant role in the therapeutic approach. Furthermore, the lack of difference in T2 biomarkers and the frequent presence of systemic comorbidities in elderly which can be negatively affected by the use of oral corticosteroids (OCS) strongly support the use of biologics, when indicated, given their steroid sparing effect.

In conclusion, our study provides some insights about potential tools for a personalized approach to patients suffering from severe asthma with particular attention to what are the clinical and instrumental data to be valued depending on age, and what clinical outcomes should be assessed as a marker of disease control and treatment response.

## Abbreviations

ASA: Acetylsalicylic Acid, ATS: American Thoracic Society, BMI: Body Mass Index, COPD: Chronic Obstructive Pulmonary Disease, ER: Emergency Room, ERS: European Respiratory Society, FeNO: Fractional Exhaled Nitric OxideF, EV1: Forced Expiratory Volume in the first second, FVC: Forced Vital Capacity, GERD: Gastroesophageal Reflux Disease, GINA: Global Initiative for Asthma, HRCT: High-Resolution Computed Tomography, IgE: Immunoglobulin E, NSAID: Non-Steroid Anti-Inflammatory Drugs, OCS: Oral Corticosteroids, OSAS: Obstructive Sleep Apnoea Syndrome, SANI: Severe Asthma Network Italy, SD: Standard Deviation.

## Funding

The Severe Asthma Network Italy (SANI) project is supported by Global Initiative for Asthma (GINA) Italy; Federasma; Italian Society of Allergy, Asthma and Clinical Immunology; and Italian Respiratory Society, through unrestricted support from AstraZeneca, GlaxoSmithKline, and Sanofi. This research received no external funding.

## Availability of data and materials

The full dataset supporting the reported results is available upon request to the corresponding author.

## Authors' contributions

AV, EMH, GG, GS, MC, MM made substantial contributions to the conception and design of the work; AM, GA, GWC, MM, PM, RV and JM, to the acquisition, analysis, and interpretation of data; AV, FB, GG, GS, MC, and PP have drafted the work or substantively revised it.

All authors approved the submitted version and agreed both to be personally accountable for the author's own contributions and to ensure that questions related to the accuracy or integrity of any part of the work.

## Ethics approval and consent to participate

The SANI registry was constructed according to the Declarations of Helsinki and Oviedo, and was set up according to the 3rd Edition Recommendation on registries for evaluating patient outcomes published by the Effective Healthcare Research and Quality. The protocol has been performed according to the Good Clinical Practice and in accordance with the Italian laws (Legislative Decree n. 211, 24 June 2003; Legislative Decree n. 200, 6 November 2007; Ministry Decree 21 December 2007). The protocol has been approved by the Local Ethical Committee of Area Vasta NORD-OVEST Toscana (Protocol Number 1245/2016, 7 December 2016) and the enrollment in the other Center started upon approval of the local ethics committees.

Informed consent was obtained from all subjects involved in the study.

## Consent for publication

All authors have approved the submission of this manuscript. The results have not been previously published and are not being considered for publication in another journal.

## Declaration of competing interest

FB received financial grants from AstraZeneca Financial, Chiesi Farmaceutici S.p.A and Insmed Inc.; he worked as a paid consultant for Menarini and Zambon; and received speaker fees from AstraZeneca, Chiesi Farmaceutici S.p.A., GlaxoSmithKline, Guidotti, Grifols, Insmed Inc., Menarini, Novartis AG, Sanofi-Genzyme, Viatris Inc., Vertex Pharmaceuticals and Zambon. MC received financial grants from AstraZeneca, GSK, Sanofi. GWC reports having received research grants as well as being lecturer or having received advisory board fees from: A. Menarini, Allergy Therapeutics, AstraZeneca, Chiesi Farmaceutici, Faes, Firma, Guidotti-Malesci, Glaxo Smith Kline, Hal Allergy, Innovacaremd, Novartis, OmPharma, RedMaple, Sanofi-Aventis, Sanofi-Genzyme, Stallergenes-Greer, Uriach Pharma, ThermoFisher, Valeas. EH received a research grant from GlaxoSmith&Kline, and fees for lectures from Sanofi, Regeneron, GlaxoSmith&Kline, Astrazeneca, Novartis, Chiesi, Stallergenes-Greer; and declares fees for advisory boards participation from Sanofi, Regeneron, Glaxo Smith Kline, Astrazeneca, Novartis, Chiesi, Almirall, Celltrion Healthcare, Bosch. PP received advisory board fees from Chiesi Farmaceutici, Glaxo Smith Kline, and Sanofi, and fees for educational activities from: AstraZeneca, Chiesi Farmaceutici, Glaxo Smith Kline, Guidotti and Sanofi. GS received financial grants from AstraZeneca, GSK, Novartis, Sanofi. AM, AV, GA, GG, JM, MM, PM and RV declared no relevant conflicts of interest.

All authors reported no financial interests or potential conflicts of interest related to this study.
